# Intestinal Anti-Inflammatory Activity of *Baccharis dracunculifolia* in the Trinitrobenzenesulphonic Acid Model of Rat Colitis

**DOI:** 10.1093/ecam/nep081

**Published:** 2011-06-23

**Authors:** Sílvia Helena Cestari, Jairo Kennup Bastos, Luiz Claudio Di Stasi

**Affiliations:** ^1^Laboratory of Phytomedicines, Department of Pharmacology, Instituto de Biociências, São Paulo State University–UNESP, Botucatu 18.618-000, São Paulo, Brazil; ^2^Faculty of Pharmaceutical Sciences, University of São Paulo, Ribeirão Preto 14040-903, São Paulo, Brazil

## Abstract

*Baccharis dracunculifolia* DC (Asteraceae) is a Brazilian medicinal plant popularly used for its antiulcer and anti-inflammatory properties. This plant is the main botanical source of Brazilian green propolis, a natural product incorporated into food and beverages to improve health. The present study aimed to investigate the chemical profile and intestinal anti-inflammatory activity of *B. dracunculifolia* extract on experimental ulcerative colitis induced by trinitrobenzenosulfonic acid (TNBS). Colonic damage was evaluated macroscopically and biochemically through its evaluation of glutathione content and its myeloperoxidase (MPO) and alkaline phosphatase activities. Additional *in vitro* experiments were performed in order to test the antioxidant activity by inhibition of induced lipid peroxidation in the rat brain membrane. Phytochemical analysis was performed by HPLC using authentic standards. The administration of plant extract (5 and 50 mg kg^−1^) significantly attenuated the colonic damage induced by TNBS as evidenced both macroscopically and biochemically. This beneficial effect can be associated with an improvement in the colonic oxidative status, since plant extract prevented glutathione depletion, inhibited lipid peroxidation and reduced MPO activity. Caffeic acid, *p*-coumaric acid, aromadendrin-4-*O*-methyl ether, 3-prenyl-*p*-coumaric acid, 3,5-diprenyl-*p*-coumaric acid and baccharin were detected in the plant extract.

## 1. Introduction

The inflammatory bowel diseases (IBD) refer essentially to two different but closely related conditions, Crohn's disease and ulcerative colitis. Although the etiology of IBD remains unclear, there is evidence that it involves immune, genetic and environmental factors, which are, in turn, related to the initiation and progression of colitis [1, 2]. IBD is related to an abnormally exacerbated immune response to otherwise innocuous stimuli which is not properly abrogated by the feedback system that normally downregulates the mucosal response to luminal factors [3]. As a consequence, increased numbers of inflammatory cells are found in areas of intestine with chronic inflammation, resulting in overproduction of variety of proinflammatory mediators including eicosanoids, platelet-activating factor, cytokines and reactive oxygen and nitrogen metabolites [1–4]. The oxidative stress through an excessive release of reactive oxygen species has been hypothesized to play a key role in IBD pathogenesis [5, 6]. In fact, antioxidant activity may be responsible for the beneficial effects shown by 5-aminosalicylates in human IBD [7] and for benefits derived from different natural compounds in experimental models, mainly phenolic compounds as flavonoids [8], tempol [9], isocoumarin [10] and coumarin derivatives [11].

*Baccharis dracunculifolia* DC (Asteraceae) is a Brazilian medicinal shrub popularly known as “Alecrim do Campo” that is used against ulcer, inflammation and hepatic disorders [12, 13]. This medicinal plant was found to be the main botanical source of resin and chemical constituents of Brazilian propolis, denominated green propolis, which was recently incorporated into food and beverages to improve health and to prevent several diseases [14–16].

In recent years, there has been growing interest in studying the chemical profile and biological activity of Brazilian green propolis and its main botanical source, *B. dracunculifolia*. Several studies have identified *B. dracunculifolia* as an important source of active compounds with trypanocidal [17], antiulcer [18], antimicrobial [19], antimutagenic [20], immunomodulatory [21], anti-inflammatory [22] and radical scavenging [23] activities. Although green propolis composition is more complex and unpredictable than previously assumed [24], it is clear that pharmacological potentialities of green propolis are related to substances previously known as plant constituents, mainly from *B*. *dracunculifolia* [14, 16, 24–26].

Therefore, the present work aimed to evaluate the intestinal anti-inflammatory activity of ethyl acetate extract from aerial parts of *B. dracunculifolia* in the trinitrobenzenesulfonic (TNBS) acid model of rat colitis. For this purpose, we assayed the effects of *B. dracunculifolia* extract in preventing the inflammatory response induced by TNBS in two different experimental settings, that is, when the colonic mucosa is intact and when the mucosa is healing after an initial insult. Thus, the present work also sought to determine the chemical constituents of the *B. dracunculifolia* ethyl acetate extract through HPLC analysis.

## 2. Methods

### 2.1. Preparation of Plant Extract

The aerial parts of *B. dracunculifolia* DC were collected in Cajuru city, State of São Paulo, Brazil, in December 2005. The plant material was authenticated by Jimi N. Nakagima, and a voucher specimen (SPFR 06143) was deposited in the Herbarium of the Biology Department of the University of São Paulo (FFCLRP) at Ribeirão Preto, State of São Paulo, Brazil. Fresh plant material was air-dried at 40°C for 48 h. The dried leaves were powdered in a blender and submitted to maceration for 24 h in ethyl acetate at room temperature. This solvent was used due to its polyphenolic content. After solvent evaporation using vacuum <40°C, an ethyl acetate extract of *B. dracunculifolia* was obtained.

### 2.2. Experimental Design

Male Wistar rats (200 ± 20 g) obtained from the Laboratory Animal Service of the São Paulo State University (UNESP) were randomly distributed in experimental groups. The study was carried out in accordance with “Guide for the Care and Use of Laboratory Animals” as promulgated by the Animal Experimental Committee of São Paulo State University (protocol number 042/04-CEEA-IB). Animals were housed in makrolon cages and maintained in air-conditioned with a 12 h light-dark cycle and air-filtration, and they were provided with free access to tap water and food. Colitis was induced by the method originally described [27]. Animals were fasted overnight and anaesthetized with halothane. Under anaesthesia, they were given 10 mg of TNBS dissolved in 0.25 ml 50% ethanol (v/v) by means of a Teflon cannula inserted 8 cm through the anus. Rats from the non-colitic group received 0.25 ml of saline.

#### 2.2.1. Acute Colitis

Rats were given 5, 10, 25, 50, 100 and 200 mg kg^−1^ per day of *B. dracunculifolia* extract for 72, 48, 24 and 2 h before colitis induction as well as 24 h thereafter. The plant extract was suspended in 1% (v/v) Tween 80 and administered by means of an oesophageal catheter. Rats from the non-colitic group were orally administered with the saline and rats non-treated colitic (TNBS-control group) with the vehicle (1% Tween 80). Animals from all groups (*n* = 6) were killed 48 h after colitis induction.

#### 2.2.2. Chronic Colitis

In this protocol, colitis was induced with 10 mg of TNBS in 50% ethanol, as previously described. The animals were divided in three groups; two groups were daily orally treated with 5 and 50 mg kg^−1^ of plant extract, one group received instead 25 mg kg^−1^ of sulfasalazine suspended in the same vehicle. Treatments started 2 h after the first administration of TNBS and continued until the day before the animals were killed. Two additional groups were included for reference: a non-colitic group and a colitic group, receiving the latter only the first dose of TNBS (TNBS control group). The animals of these groups were given the vehicle (5 ml kg^−1^ 1% Tween 80) orally. Animals from each group were killed after 1 or 2 weeks of the colitis induction.

#### 2.2.3. Assessment of Colonic Damage

Animal body weights, occurrence of diarrhoea and adherence of adjacent organs were recorded. The animals were euthanized by an overdose of halothane, and colonic segments were obtained after laparotomy. Colon were placed on an ice-cold plate, cleaned of fat and mesentery, and blotted on filter paper, weighed and its length measured under a constant load (2 g). The colon was longitudinally opened and scored for macroscopically visible damage on a 0–10 scale by two observers unaware of the treatment, according to the criteria described [28] in Table 1. The colon was subsequently divided longitudinally into different pieces to be used for the biochemical determinations: total glutathione (GSH) content [29], myeloperoxidase (MPO) activity [30] and alkaline phosphatase (AP) activity [31].

Additional *in vitro* experiments were performed in order to test the antioxidant activity of different concentrations of the ethyl acetate of *B. dracunculifolia* (12.5–1600 *μ*g ml^−1^). These were evaluated by the assay of lipid peroxidation in the rat brain membrane modified from the original protocol [32]. Briefly, rat brain samples were obtained from 4-month-old male Wistar rats. All samples were diluted upto 1 : 10 (w/v) in a buffer containing (mmol l^−1^) Tris 50.0, NaCl 100, KCl 0.5, CaCl_2_ 0.5, MgSO_4_ 1.0, KH_2_PO_4_ 0.55 and sucrose (pH 7.4) for preparation of a membrane-enriched fraction. This membrane concentration was diluted upto 1 : 4 v/v in the above-mentioned buffer solution but with 20 mol l^−1^ Tris. Then, buffer (in the assays without inhibitors) or different concentrations of plant extract were added. Lipid peroxidation was induced via nonenzymatic way with 100 *μ*m ol l^−1^ of both ferrous sulfate and ascorbic acid. After incubation at 37°C for 45 min, the reaction was stopped and malondialdehyde (MDA) was analysed using 0.5% thiobarbituric acid in 20% trichloroacetic acid. The amount of MDA produced after agitation, incubation at 100°C for 15 min and centrifugation at 1000 g for 15 min at 4°C, was determined by measuring the spectrophotometric absorbance of the supernatant at 532 nm. The flavonoid quercetin was used as reference and tested in the same assay system.

### 2.3. Statistical Analysis

All results are expressed as the mean ± SEM. Differences between means were tested for statistical significance using one-way analysis of variance (ANOVA) and *post hoc* least significance tests. Nonparametric data (score) are expressed as median (range) and were analysed with the Kruskal–Wallis test. Statistical significance was set at *P* < .05.

### 2.4. Phytochemical Analysis of Plant Extract by HPLC

The ethyl acetate extract of the aerial parts from *B. dracunculifolia* was submitted to HPLC analysis using the following equipment and conditions: a Shimadzu high performance liquid-chromatograph (SCL-10A*vp* system controller, three LC-10AD pumps, SPD-M10A*vp* photodiode array detector and Shimadzu Class-VP 5.02 software). A CLC-ODS (M) column (4.6 mm i.d. × 250 mm, 5 *μ*m particle diameter) and a CLC G-ODS guard column were used as the stationary phase. The mobile phase had the following composition: A–B: 25–100% (B) in 60 min, A: 93.9% water, 0.8% acetic acid, 0.3% ammonium acetate, 5.0% methanol; B: acetonitrile; detection: 280 nm, flow rate of 1 ml min^−1^. The compounds detected were identified by comparison with authentic standards available, comparing UV spectra and considering both the maximum lambda and the relative area obtained with the use of two wavelengths (A280/320). The ethyl acetate extract was dissolved in methanol (HPLC grade) to obtain a concentration of 1 mg ml^−1^. Before analysis, all samples were centrifuged at 1300 rpm and filtered through a 45 *μ*m filter.

## 3. Results

Administration of TNBS/ethanol resulted in colonic inflammation that presented several mucosal necrosis extending along the colon accompanied by bowel wall thickening, hyperemia and focal adhesions to adjacent organs (Table 2). This inflammatory process was associated with an increase of the colonic weight/length ratio and with signs of diarrhea in 80% of the colitic animals (Table 2). In addition, the colonic MPO and AP activities increased by 8-fold and 2-fold, respectively, whereas glutathione levels were reduced by 56% in comparison with non-colitic rats (Table 3).

Oral administration of the plant extract at the doses of 5 and 50 mg kg^−1^ significantly attenuated the macroscopic mucosal damage in the TNBS/ethanol model of colitis in rats (Table 2). Administration of 5 mg kg^−1^ of the *B. dracunculifolia* extract to colitic animals was able to counteract the colonic glutathione depletion that took place as a result of the colonic oxidative damage induced by TNBS (Table 3). Colonic tissue's MPO activity differed significantly between controls and treated colitic rats at the doses 5, 50, 100 and 200 mg kg^−1^ (Table 3). AP activity was also reduced in rats treated with 50 mg kg^−1^ of plant extract. The effects of plant extract were similar to those produced by sulfasalazine (Tables 2 and 3). These results prompt us to select both doses (5 and 50 mg kg^−1^) for testing with the chronic protocol.

In the chronic colitis, the inflammatory process induced by intracolonic instillation of 10 mg of TNBS in 50% ethanol (v/v) progressed over time with characteristics similar to those previously reported [10, 11]. Indeed, an alteration of the colonic absorptive function was noted since 67% of rats evidenced signs of diarrhea (Table 4).

Administration of 5 and 50 mg kg^−1^ of *B. dracunculifolia* extract to colitic animals reduced damage score, lesion extension, diarrhoea signs and adherence of colon to adjacent organs after the first week (Table 4). Thus, both doses of *B. dracunculifolia* extract were able to counteract GSH content and to reduce MPO and AP activities by the end of the first week (Table 5). After 2 weeks, both doses of plant extract were actively counteracting the GSH content and reducing AP activity (Table 5).

The *in vitro* experiments performed showed that the plant extract exerts an inhibitory effect on the lipid peroxidation induced in rat brain membranes, with an IC_50_ value of 26.33 *μ*g ml^−1^. The corresponding IC_50_ value of quercetin was 1.51 *μ*m corresponding to 0.46 *μ*g ml^−1^.

**Figure 1:**
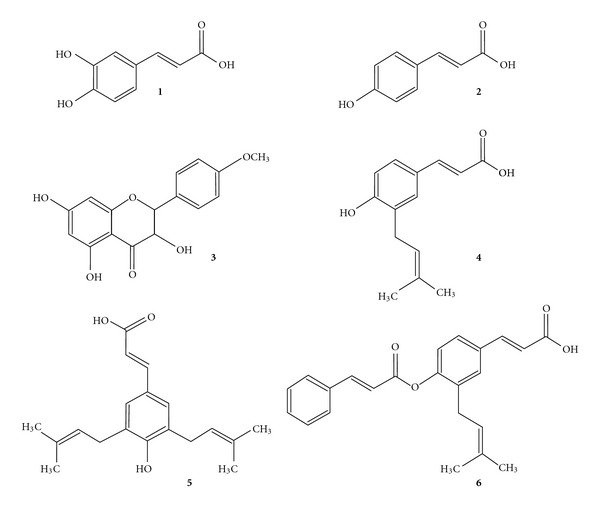
Structures of chemical constituents in the ethyl acetate extract of *B. dracunculifolia*. (**1**) caffeic acid; (**2**) *p*-coumaric acid; (**3**) aromadendrin-4-*O*-methyl ether; (**4**) 3-prenyl-*p*-coumaric acid (drupanin); (**5**) 3,5-diprenyl *p*-coumaric acid (artepillin C); (**6**) baccharin.

**Figure 2:**
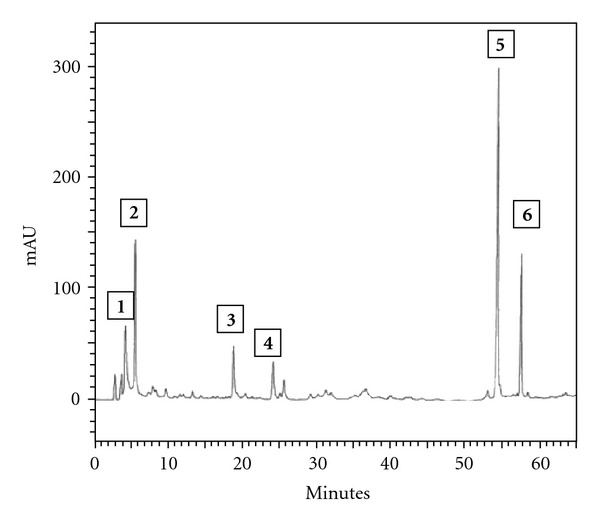
HPLC profile of *B. dracunculifolia*: (**1**) caffeic acid; (**2**) *p*-coumaric acid; (**3**) aromadendrin-4-*O*-methyl ether; (**4**) 3-prenyl-*p*-coumaric acid (drupanin); (**5**) 3,5-diprenyl *p*-coumaric acid (artepillin C); (**6**) baccharin.

HPLC analysis of the ethyl acetate extract of *B. dracunculifolia* permitted the identification of only the following compounds: caffeic acid, *p*-coumaric acid, aromadendrin-4-*O*-methyl ether, 3-prenyl-*p*-coumaric acid, 3,5-diprenyl-*p*-coumaric acid and baccharin (Figures 1 and 2).

## 4. Discussion

The role of reactive metabolites of oxygen and nitrogen in the pathophysiology of IBD has been well reported in humans as experimental models of colitis [6, 10, 33, 34]. The inflammatory process in the intestine is probably derived from the chronic presence of numerous, activated, MPO-containing phagocytes in the inflamed intestine. These are responsible for the overproduction of reactive oxygen species that overwhelm the antioxidant defences, such as glutathione and its related enzymes that normally participle in protecting colonic tissue from oxidative damage. In fact, it has been reported that the production of reactive oxygen species is considerably increased in colonic biopsy specimens from ulcerative colitis and Crohn's disease patients in comparison with normal control mucosa, in a manner positively correlated with inflammatory bowel disease activity. This phenomenon appears to be neutrophil derived [35]. Thus, antioxidant therapy can constitute an interesting approach in the downregulation of this inflammatory condition. In the context of this effect, the beneficial protection exerted by the 5-aminosalicylic derivatives has been attributed to their antioxidant and free radical scavenger properties [7]. Thus, plant extracts containing antioxidant compounds such as flavonoids and other phenolic compounds may be of prime interest [36].

The present study shows the preventive effect exerted by an ethyl acetate extract of *B. dracunculifolia* in ameliorating the colonic insult induced after intracolonic administration of TNBS to rats. The preventive intestinal anti-inflammatory activity of this plant extract (5 and 50 mg kg^−1^) was evidenced macroscopically by reducing the damage score of the lesions in the acute phase of the TNBS-induced inflammatory process. Similar effects were produced by oral administration of 25 mg kg^−1^ of sulfasalazine, a drug of choice for treating IBD. Both *B. dracunculifolia* extract and sulfasalazine failed to decrease the colonic weight/length ratio. The lack of effect on this ratio can be explained by the severe and extensive colonic damage induced by TNBS/ethanol, which is difficult to overcome by pharmacological treatment as has been previously suggested [37]. In the acute phase of the colonic inflammatory process, the protective effects of *B. dracunculifolia* extract were not dose dependent, since higher doses of plant extract resulted in the loss of activity. A dual effect has already been described for flavonoids, such as diosmin and hesperidin [38], quercetin [33] and silymarin [39], isocoumarin paepalantine [10] as well as coumarin and 4-hydroxycoumarin [11], and may be related to the known capacity of these compounds to behave as antioxidants at lower doses and pro-oxidants at high doses in rat colon. On the other hand, inflammation is a multicomponent system that involves a network of cellular crosstalk and control. However, the anti-inflammatory effect produced by *B. dracunculifolia* may be related to distinct mechanisms of these compounds acting on distinct inflammatory mediators.

MPO activity has been widely used to detect and monitor intestinal inflammation, so that a reduction in the activity of this enzyme can be interpreted as a manifestation of the anti-inflammatory property of a given compound [36, 40]. Similarly, AP activity can also be considered as a sensitive marker of inflammation in the intestine, since this enzyme's activity is invariably augmented in experimental conditions [32, 41]. One of these mechanisms could be the antioxidant properties of *B. dracunculifolia* extract. This property may play a crucial role in the intestinal anti-inflammatory effect of the plant extract, given that intense oxidative insult is a common feature in human IBD [42, 43] and in the different experimental models of rat colitis including TNBS [32, 44, 45]. In this aspect, in the present study, *B. dracunculifolia* extract treatment of TNBS colitic rats counteracted the depletion of colonic glutathione levels that took place in control colitic animals in both experimental treatment protocols. The effect exerted by *B. dracunculifolia* extract in preserving the colonic mucosa from oxidative insult may collaborate to decrease the neutrophil infiltration that occurs in response to TNBS. Free radical generation has been proposed as playing an important early role in the pathogenesis of IBD [6], and could contribute to the initial neutrophil infiltration in the inflamed colonic mucosa. The recruitment and activation of these cells result in an increase in free radical production that overwhelms the tissue's antioxidant protective mechanisms, resulting in a situation of oxidative stress, which definitively perpetuates colonic inflammation [42]. As a consequence, a rapid inhibition of free radical generation could contribute to a lower level of leukocyte infiltration into the inflamed tissue, thus preventing colonic tissue becoming inflamed. The antioxidant properties ascribed to this *B. dracunculifolia* extract could also contribute to its intestinal anti-inflammatory effect, similarly to other reputed drugs used in the treatment of IBD, such as 5-aminosalicylic derivates [46], thus supporting additional studies aiming to evaluate the application of this plant extract in the treatment of human IBD.

In the second set of experiments, the effects of *B. dracunculifolia* were evaluated after induction of the inflammatory process. The results demonstrated that oral administration of *B. dracunculifolia* extract (5 and 50 mg kg^−1^) after colitis induction significantly reduced the macroscopic colonic damage score and lesion extension after the first week when compared to the corresponding TNBS control group. In addition, these compounds were able to alter the extent of neutrophil infiltration into the colon 1 week after TNBS administration, as determined by tissue MPO activity reduction. The reduction of neutrophil infiltration seemed to be a consequence of the accelerated healing of colonic ulcers, thus facilitating the elimination of neutrophil accumulation from the inflamed colon. Similarly to the acute experimental setting, the anti-inflammatory effect exerted by *B. dracunculifolia* extract at doses of 5 and 50 mg kg^−1^ in chronic colitis was related to the protective effect against GSH depletion and inhibition of AP activities in the first and second week.

HPLC analysis of the ethyl acetate extract of *B. dracunculifolia* permitted the identification of such cinnamic acid derivatives such as caffeic, *p*-coumaric, 3-prenyl-*p*-coumaric (drupanin), 3,5-diprenyl-*p*-coumaric (artepillin C) and 3-prenyl-4-dihydrocinnamoiloxy–cinnamic (baccharin) acids, as well as the flavonoid aromadendrin-4-*O*-methyl ether (Figure 2). Artepillin C was detected as a major component of *B. dracunculifolia*. This compound is also the main constituent in Brazilian green propolis and an efficient radical scavenger [21]. Artepillin C is a potent anti-inflammatory compound that decreases the number of neutrophils during peritonitis and reduces the prostaglandin E_2_ level, nitric oxide production and NF-*κ*B activity [22]. In addition, artepillin C also suppresses the formation of aberrant crypt foci induced by azoxymethane in mouse colon after oral administration [47]. Phenolic compounds including cinnamic acid derivatives detected in *B. dracunculifolia* extract have been identified as responsible for antiulcerogenic [18], immunomodulatory [21], antitumoural [48, 49], neuroprotective and antioxidant [23, 50] activities. Polyphenolic compounds of higher plants are known to be excellent *in vitro* antioxidants, while numerous studies suggest that dietary intake of plant phenolics may produce positive effects on oxidative stress-related diseases [51]. The compounds of *B. dracunculifolia* presented free radical scavenging activity similar to that of green propolis, and phenolic compounds are found abundantly in both [52].

**Figure 3:**
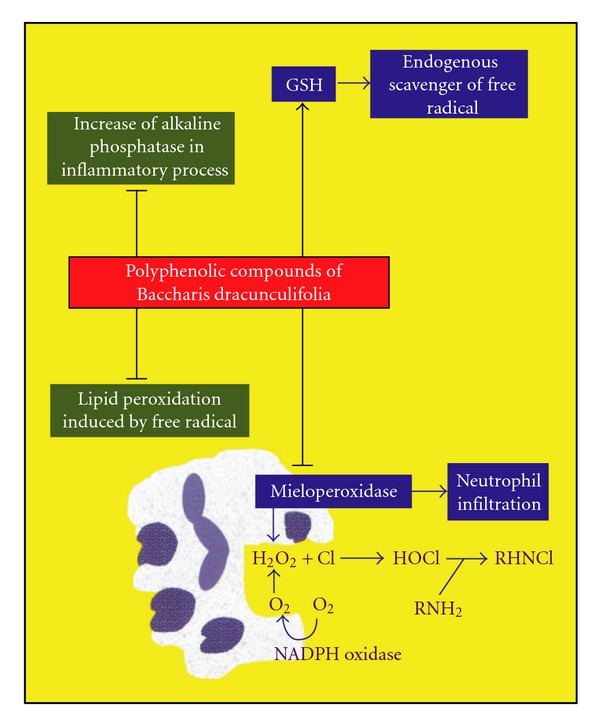
Main effects of the *B. dracunculifolia* on the inflammatory intestinal process induced by TNBS in rats. Black lines indicate inhibition and black arrows indicate counteraction of depletion induced by the inflammatory process.

In conclusion, the present study showed that *B. dracunculifolia* prevents colonic damage induced by TNBS in rats with either acute or chronic colitis. This anti-inflammatory effect may be associated with improved intestinal oxidative stress (Figure 3), mainly due to reduced MPO activity, augmented endogenous antioxidant defences in the inflamed colon, such as glutathione content and inhibited lipid peroxidation. This intestinal anti-inflammatory activity is related to the phenolic compounds present in the plant extract, mainly artepillin C. In this manner, the use of *B. dracunculifolia*, the main source of active compounds in Brazilian green propolis, as a dietary product, can be an important supplement in the treatment and prevention of human IBD.

## Funding

CNPq (Science and Technology Ministry, Brazil); CAPES (Coordenação de Aperfeiçoamento de Pessoal de Nível Superior, Brazilian Ministry of Education); and FAPESP (Fundação de Amparo à Pesquisa do Estado de São Paulo).

## Figures and Tables

**Table 1:** Criteria for assessment of macroscopic colonic damage

Score	Criteria
0	No damage
1	Hyperemia, no ulcers
2	Linear ulcer with no significant inflammation
3	Linear ulcer with inflammation at one site
4	Two or more sites of ulceration/inflammation
5	Two or more major sites of ulceration and inflammation or one site of ulceration/inflammation extending >1 cm along the length of the colon
6–10	If damage covers >2 cm along the length of the colon, the score is increased by 1 for each additional centimeter of involvement

**Table 2:** Effects of *B. dracunculifolia* extract (5–200 mg kg^−1^) and sulfasalazine (25 mg kg^−1^) on damage score, changes in colonic weight/length, diarrhea and adherence incidence in TNBS acute colitis

Group	Damage score^a^ (0–10)	Colonic weight/length^b^ (mg cm^−1^)	Diarrhoea (%)	Adherence (%)
Non-colitic	0***	82.30 ± 3.86**	0	0
TNBS-control	8.5 (7–9)	146.99 ± 8.78	80.0	50.0
*B. dracunculifolia*			
5 mg kg^−1^	6.0 (5–8)*	127.44 ± 8.06	50.0	50.0
10 mg kg^−1^	7.5 (6–8)	127.82 ± 3.94	50.0	66.7
25 mg kg^−1^	7.5 (5–10)	135.28 ± 6.33	66.7	66.7
50 mg kg^−1^	6.5 (5–8)*	139.10 ± 9.05	33.3*	0*
100 mg kg^−1^	6.5 (4–8)	131.53 ± 7.34	83.3	33.3
200 mg kg^−1^	7.0 (4–8)	134.62 ± 9.11	50.0	33.3
Sulfasalazine	6.0 (2–8)**	133.58 ± 12.38	16.7	50.0

^a^Score data are expressed as median (range).

^b^Colonic weight data are expressed as mean ± SEM.

*P < .05; **P < .01; ***P < .001 versus TNBS control group.

**Table 3:** Effects of *B. dracunculifolia* extract (5–200 mg kg^−1^) and sulfasalazine (25 mg kg^−1^) on GSH content, MPO activity and AP activity in TNBS acute colitis

Group	GSH content (nmol g^−1^ tissue)	MPO activity (U g^−1^ tissue)	AP activity (mU mg^−1^ protein)
Non-colitic	1357.5 ± 86.5**	84.2 ± 8.7**	6.24 ± 0.36*
TNBS control	759.6 ± 31.9	684.2 ± 62.8	12.62 ± 1.72
*B. dracunculifolia*		
5 mg kg^−1^	967.3 ± 55.0*	324.9 ± 89.9**	10.53 ± 1.21
10 mg kg^−1^	850.3 ± 27.8	532.8 ± 96.3	14.58 ± 1.36
25 mg kg^−1^	878.5 ± 82.7	576.4 ± 79.8	14.27 ± 2.02
50 mg kg^−1^	809.4 ± 55.0	216.9 ± 31.6**	6.16 ± 0.63*
100 mg/kg^−1^	873.8 ± 25.8	245.8 ± 39.9**	7.45 ± 0.93
200 mg kg^−1^	930.9 ± 113.8	380.1 ± 84.4*	9.54 ± 1.76
Sulfasalazine	1006.2 ± 87.0*	419.8 ± 80.1*	9.02 ± 1.56

Data are expressed as mean ± SEM. *P < .05, **P < .01 versus TNBS control group.

**Table 4:** Effects of *B. dracunculifolia* extract (5 and 50 mg kg^−1^) and sulfasalazine (25 mg kg^−1^) on damage score, changes in colonic weight/length, diarrhea and adherence incidence in TNBS chronic colitis with relapse

Group	Damage score^a^ (0–10)	Colonic weight/lenght^b^ (mg cm^−1^)	Diarrhoea (%)	Adherence (%)
1 week			
Non-colitic	0***	100.57 ± 8.03**	0*
TNBS-control	8.0 (7–9)	276.10 ± 31.17	83.3
* B. dracunculifolia* 5 mg kg^−1^	2.0 (0–4)*	140.53 ± 8.61**	0*
* B. dracunculifolia* 50 mg kg^−1^	1.0 (0–5)*	171.00 ± 22.91**	33.3
Sulfasalazine 25 mg kg^−1^	3.5 (2–7)	143.25 ± 20.56**	0
2 week			
Non-colitic	0***	87.16 ± 4.32**	0
TNBS-control	2.5 (2–3)	185.40 ± 22.21	16.7
* B. dracunculifolia* 5 mg kg^−1^	3.0 (0–4)	117.50 ± 6.45**	0
* B. dracunculifolia* 50 mg kg^−1^	4.0 (2–5)	141.90 ± 12.02	33.3
Sulfasalazine 25 mg kg^−1^	4.0 (1–6)	137.95 ± 10.73	0

^a^Score data are expressed as median (range).

^b^Colonic weight data are expressed as mean ± SEM.

*P < .05, **P < .01, ***P < .001 versus TNBS control group.

**Table 5:** Effects of *B. dracunculifolia* extract (5 and 50 mg kg^−1^) and sulfasalazine (25 mg kg^−1^) on GSH content, MPO activity and AP activity in TNBS chronic colitis with relapse

Group	GSH content (nmol g^−1^ tissue)	MPO activity (U g^−1^ tissue)	AP activity (mU mg^−1^ protein)
1 week			
Non-colitic	2357.6 ± 311.3*	127.6 ± 14.5**	4.20 ± 0.36**
TNBS control	1590.1 ± 53.6	1045.2 ± 231.2	19.15 ± 4.38
* B. dracunculifolia* 5 mg kg^−1^	2386.1 ± 177.1*	221.1 ± 59.2*	7.93 ± 1.13**
* B. dracunculifolia* 50 mg kg^−1^	2218.6 ± 97.4*	167.6 ± 12.7*	8.31 ± 1.51**
Sulfasalazine 25 mg kg^−1^	1992.2 ± 254.2	951.0 ± 328.0	7.45 ± 0.92**
2 week			
Non-colitic	2233.9 ± 192.5	115.9 ± 12.9	5.79 ± 0.70**
TNBS control	1881.3 ± 146.8	220.6 ± 42.9	13.31 ± 1.65
* B. dracunculifolia* 5 mg kg^−1^	2228.3 ± 132.7*	167.5 ± 31.7	8.39 ± 1.07*
* B. dracunculifolia* 50 mg kg^−1^	2538.2 ± 132.8**	178.5 ± 34.6	8.78 ± 1.13*
Sulfasalazine 25 mg kg^−1^	1776.6 ± 63.5	262.4 ± 59.4	11.20 ± 1.29

Data are expressed as mean ± SEM.

*P < .05, **P < .01 versus TNBS control group.
